# Supplementary material for: Viral Metagenomics Reveals a Putative Novel HPV Type in Anogenital Wart Tissues
Source: Pathogens. 2022 Dec 1;11(12):1452. doi: 10.3390/pathogens11121452 (PMC9781650; doi:10.3390/pathogens11121452)
Supplement: Supplementary file 1 [file pathogens-11-01452-s001.zip › Supplementary Table S1.pdf]

Supplementary Table S1. The other HPV types identified in the 110 wart samples

Genus	Species	Serotype
Alphapapillomavirus	Alphapapillomavirus 1	HPV32
	Alphapapillomavirus 3	HPV62, HPV81, HPV84
	Alphapapillomavirus 4	HPV27b, HPV57b, HPV57c
	Alphapapillomavirus 5	
	Alphapapillomavirus 6	HPV39, HPV53
	Alphapapillomavirus 7	HPV45, HPV59, HPV68b
	Alphapapillomavirus 8	HPV7, HPV40, HPV43, HPV91
	Alphapapillomavirus 9	HPV31, HPV35, HPV52, HPV58, HPV67
	Alphapapillomavirus 10	HPV6, HPV11, HPV44, HPV74
	Alphapapillomavirus 14	HPV90
Gammapapillomavirus	Gammapapillomavirus 6	HPV108
