# Supplementary figures and images for: Viral Metagenomics Reveals a Putative Novel HPV Type in Anogenital Wart Tissues
Source: Pathogens. 2022 Dec 1;11(12):1452. doi: 10.3390/pathogens11121452 (PMC9781650; doi:10.3390/pathogens11121452)

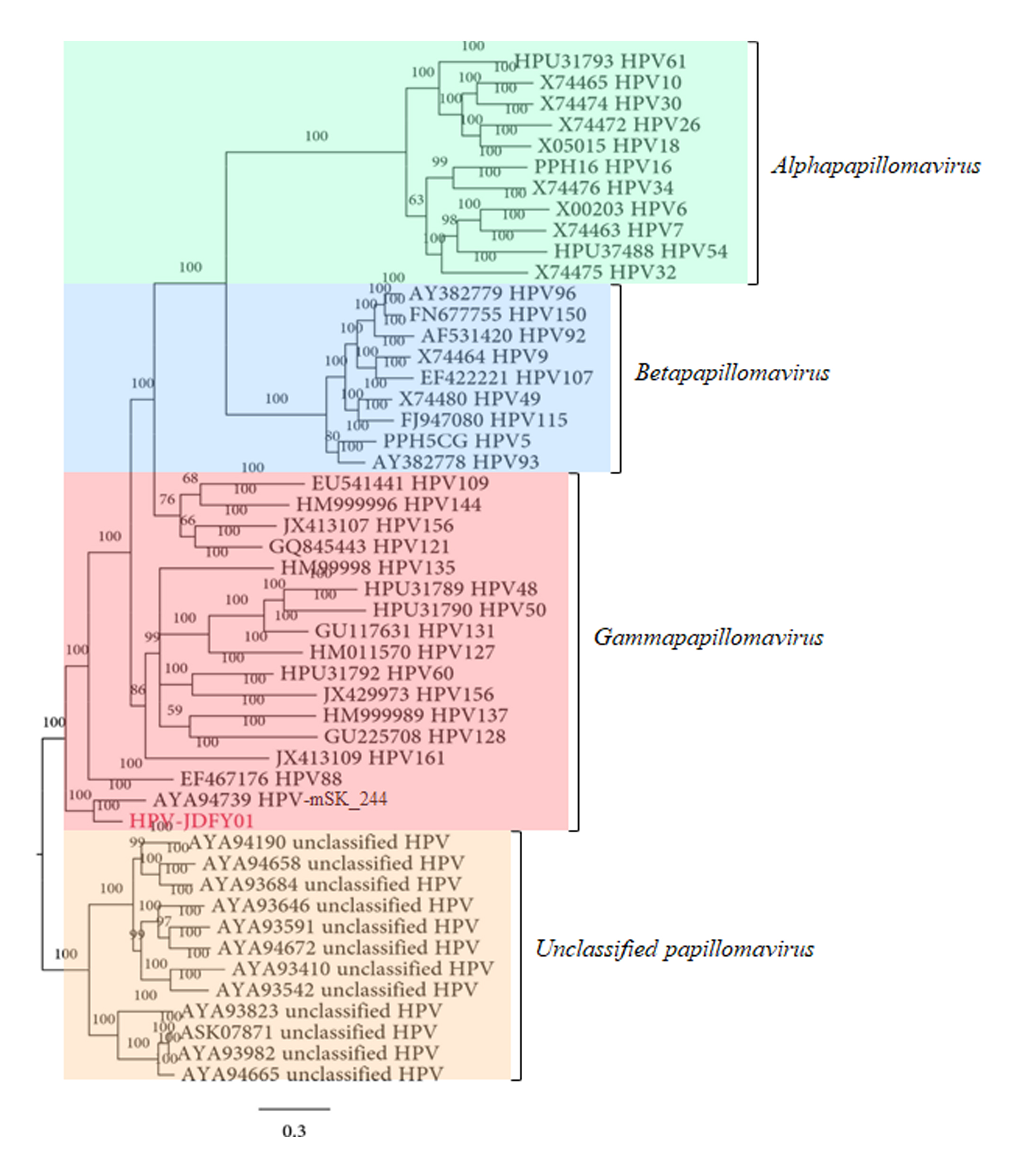

Supplement: Supplementary file 1 [file pathogens-11-01452-s001.zip › Supplementary Figure S1.jpg]

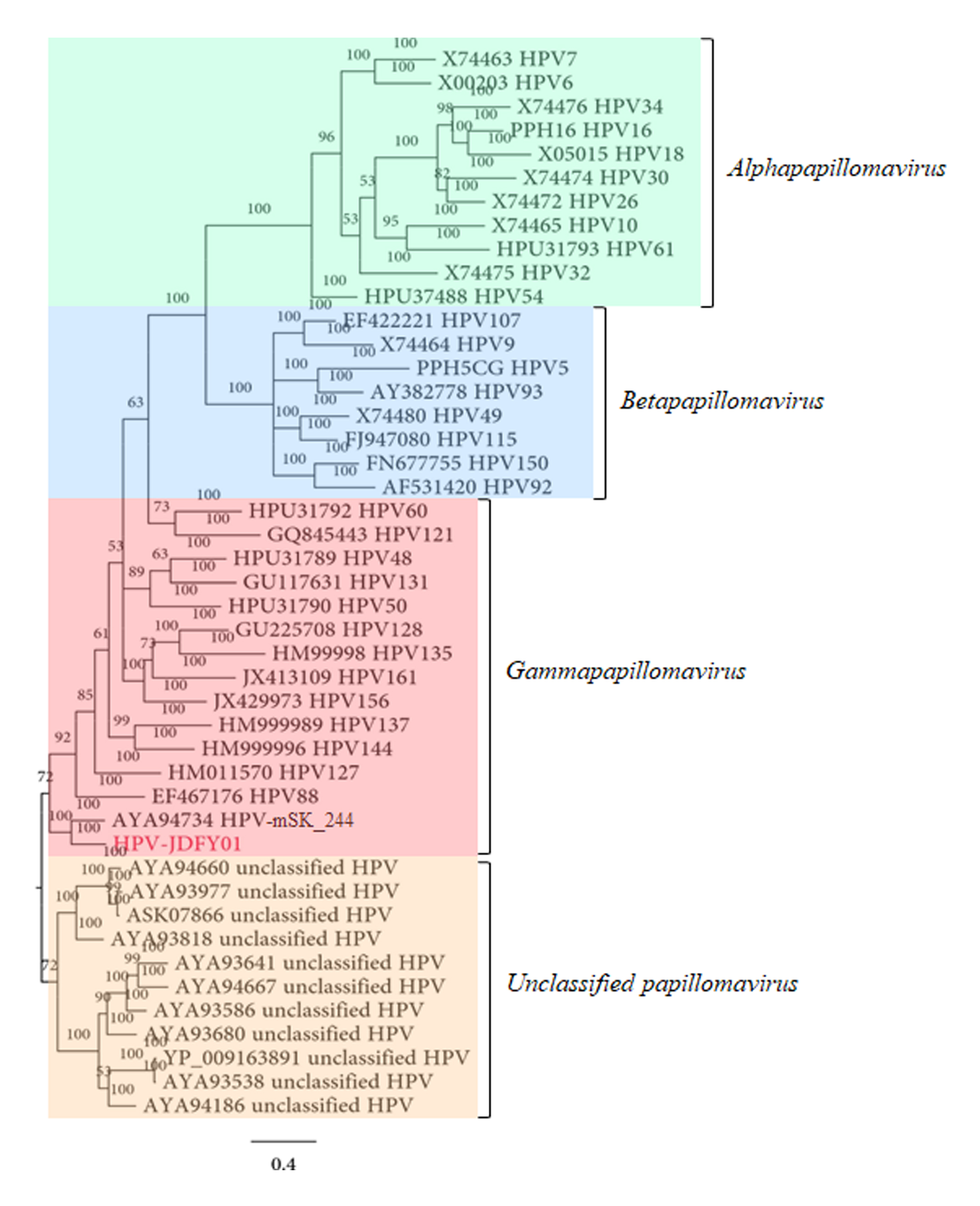

Supplement: Supplementary file 1 [file pathogens-11-01452-s001.zip › Supplementary Figure S2.jpg]

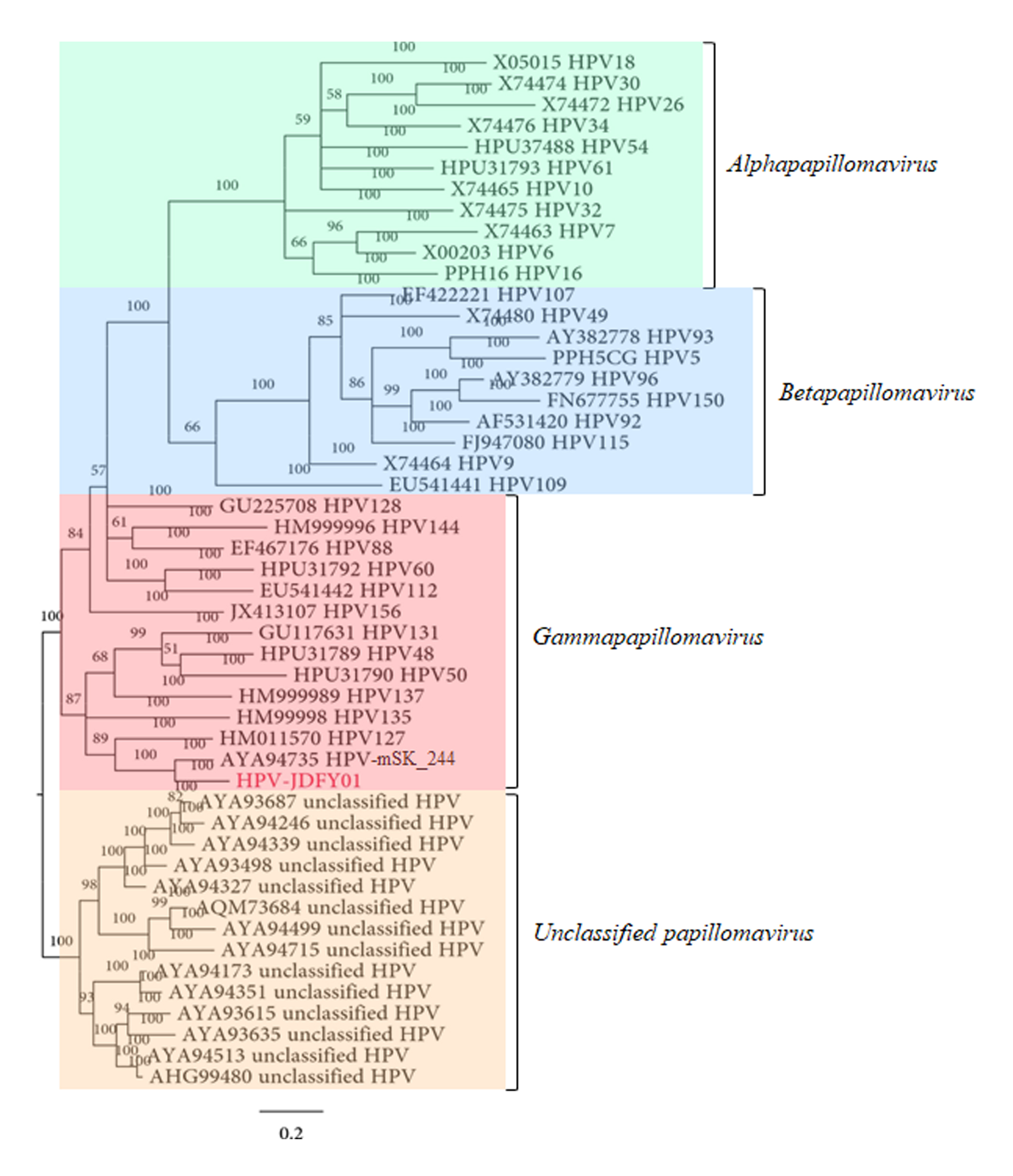

Supplement: Supplementary file 1 [file pathogens-11-01452-s001.zip › Supplementary Figure S3.jpg]

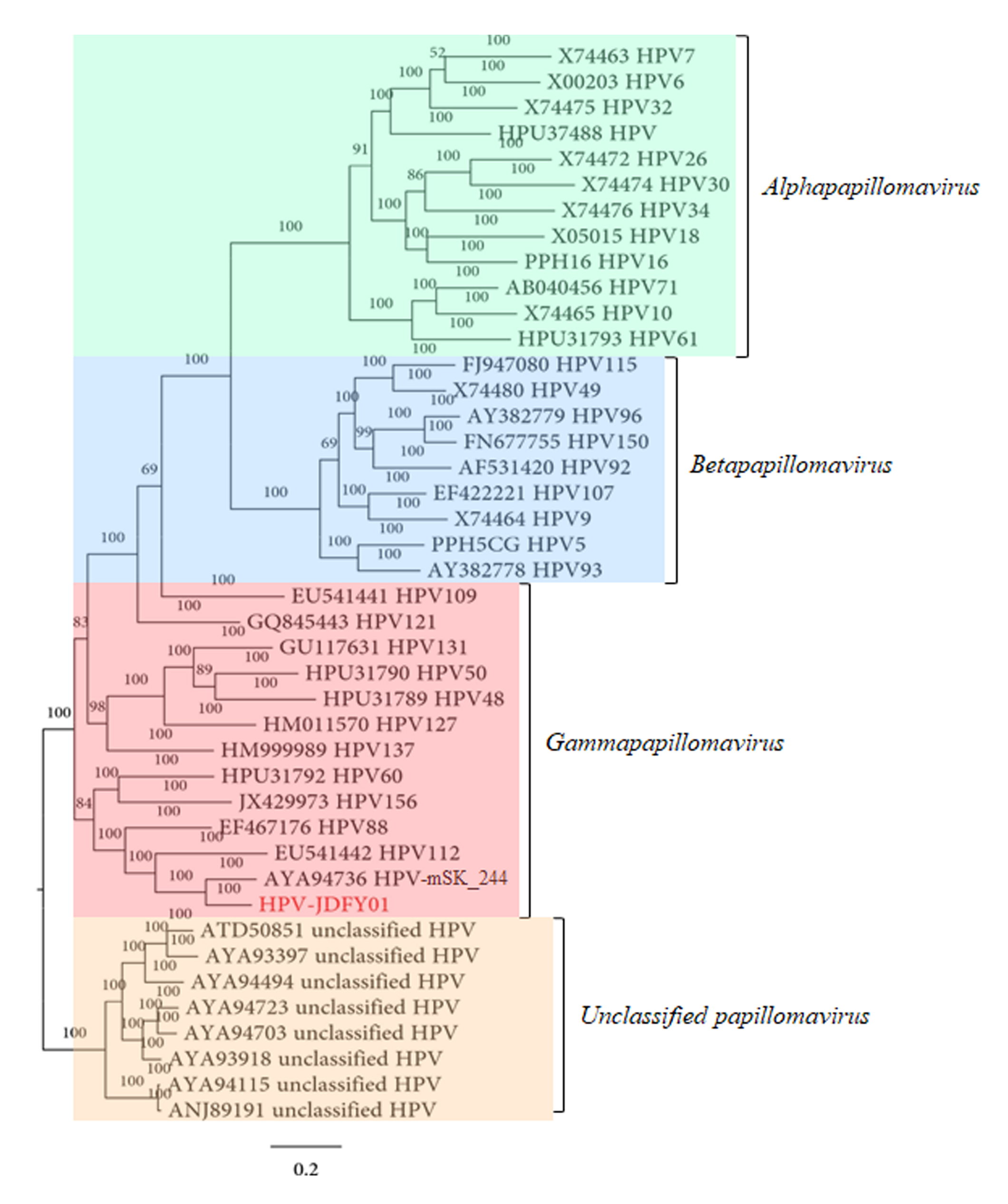

Supplement: Supplementary file 1 [file pathogens-11-01452-s001.zip › Supplementary Figure S4.jpg]

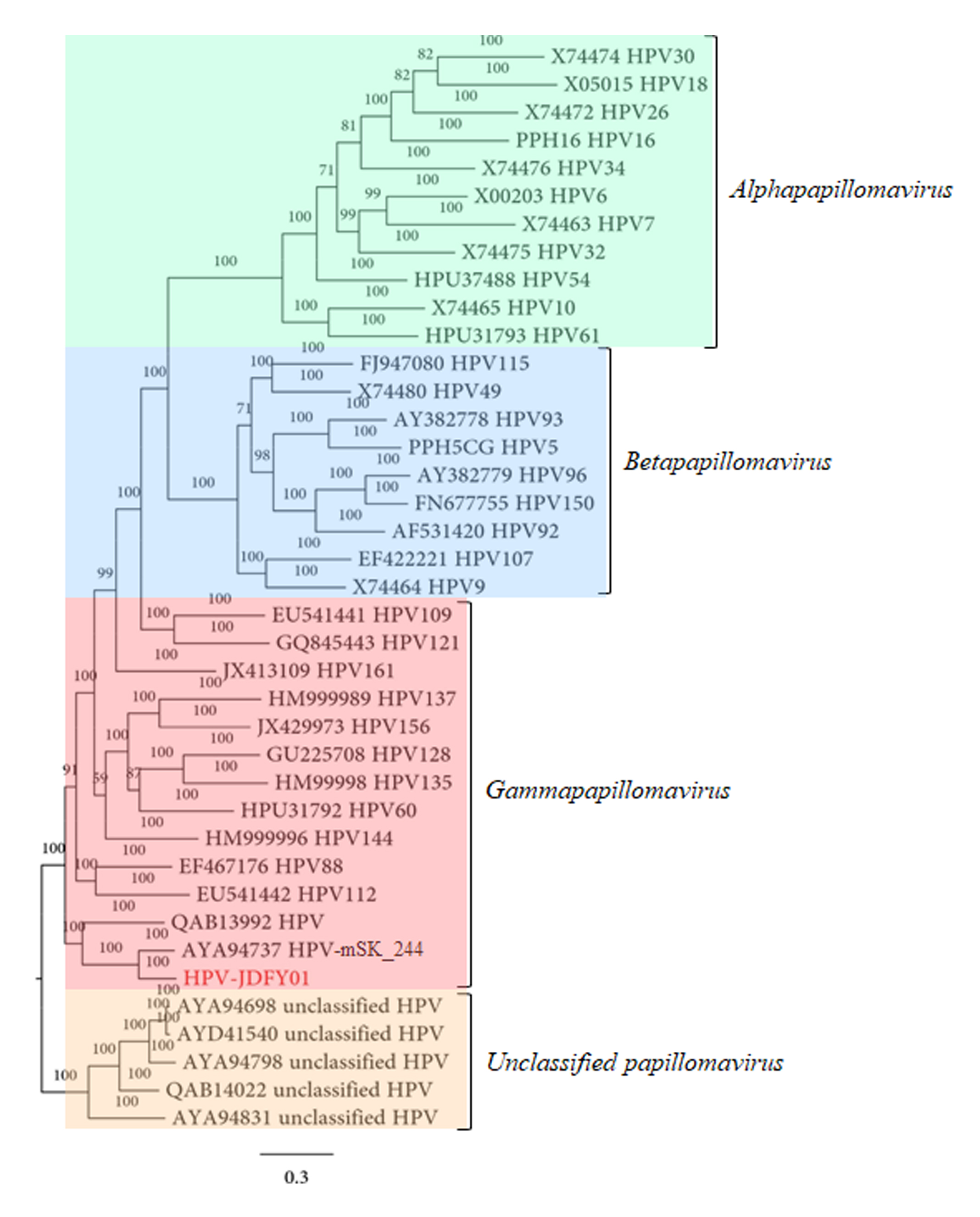

Supplement: Supplementary file 1 [file pathogens-11-01452-s001.zip › Supplementary Figure S5.jpg]
